# The step from a voluntary to a mandatory national nosocomial infection surveillance system: the influence on infection rates and surveillance effect

**DOI:** 10.1186/2047-2994-1-24

**Published:** 2012-06-08

**Authors:** Frank Schwab, Petra Gastmeier, Brar Piening, Christine Geffers

**Affiliations:** 1 National Reference Center for Surveillance of Nosocomial Infections, Berlin, Germany; 2 Institute for Hygiene and Environmental Medicine, Charité - University Medicine, Berlin, Germany

**Keywords:** Surveillance, Nosocomial infections, Neonatal intensive care unit, Bloodstream infection

## Abstract

**Abstract:**

**Background:**

The German national nosocomial infection surveillance system, KISS, has a component for very low birth weight (VLBW) infants (called NEO-KISS) which changed from a system with voluntary participation and confidential data feedback to a system with mandatory participation and confidential feedback.

**Methods:**

In order to compare voluntary and mandatory surveillance data, two groups were defined by the surveillance start date. Neonatal intensive care unit (NICU) parameters and infection rates of the NICUs in both groups were compared. In order to analyze the surveillance effect on primary bloodstream infection rates (BSI), all VLBW infants within the first three years of participation in both groups were considered. The adjusted effect measures for the year of participation were calculated.

**Results:**

An increase from 49 NICUs participating in 2005 to 152 in 2006 was observed after the introduction of mandatory participation. A total of 4280 VLBW infants was included in this analysis. Healthcare-associated incidence densities rates were similar in both groups. Using multivariate analysis with the endpoint primary BSI rate and comparing the first and third year of participation lead to an adjusted incidence rate ratio (IRR) of 0.78 (CI95 0.66-0.93) for old (voluntary) and 0.81 (CI95 0.68-0.97) for new (mandatory) participants.

**Conclusions:**

The step from a voluntary to a mandatory HCAI surveillance system alone may lead to substantial improvements on a countrywide scale.

## 

Benchmarking of healthcare associated infections (HCAI) surveillance data has been used for many years in many countries to inform preventive strategies and reduce infection rates. Most national surveillance systems were started on a voluntary basis and with confidential data feedback to the participating hospitals, but due to huge media and patient interest, mandatory participation and public reporting of HCAI have been meanwhile implemented in many countries.

The German national nosocomial infection surveillance system (KISS) was established in 1997, using the example of the U.S. National Nosocomial Infections Surveillance (NNIS) system, and focusing on ICU and post-surgery patients. As in the NNIS system, KISS was set up with voluntary participation and confidential data feedback to participating units. In 2000, a further surveillance component for very low birth weight (VLBW) infants was established (NEO-KISS), which is also on the basis of voluntary participation and confidential data feedback. It focuses on primary bloodstream infections (BSI) and pneumonia. BSI and pneumonia rates are standardized according to device use and stratified by 3 birth weight categories (< 500 g, 500–999 g, 1000–1499 g).

However, five years later, in September 2005, the “Gemeinsamer Bundesausschuss”, a joint committee of German healthcare providers and health insurance companies, required participation by all neonatal departments caring for VLBW on a mandatory basis in NEO-KISS in order to receive reimbursement. Public reporting of infection rates was not required. From the viewpoint of the organizers of NEO-KISS, it was interesting to investigate how this modification would influence infection rates and the use of surveillance data for reduction of nosocomial infections. In particular, two questions should be addressed:

Would mandatory instead of voluntary participation in the surveillance system lead to lower sensitivity in diagnosing HCAI and therefore to lower reference data with a lower value for benchmarking?

Would mandatory instead of voluntary participation lead to less intensive use of surveillance data to improve HCAI rates in individual units?

## Methods

NEO-KISS is a patient-based surveillance method for VLBW that includes patients in surveillance until a weight of 1800 g is achieved, if they do not die or are transferred earlier. The detailed surveillance method used in NEO-KISS is described elsewhere [1], [2]. It can also be found under http://www.nrz-hygiene.de/en/surveillance/hospital-infection-surveillance-system/neo-kiss/ together with the latest reference data. The following variables were collected for all patients: birth weight, sex, multiple birth, gestational age and type of delivery. Cases of primary bloodstream infections (BSI) and pneumonia were determined using modified CDC definitions [2]. Surveillance persons from each neonatal intensive care unit (NICU) have to attend an introductory course before starting data collection where the definitions are explained and trained with case studies.

### Definitions and selection criteria for analyzed NICUs

In order to compare voluntary and mandatory surveillance data, two groups were defined by the surveillance start date of the first patient within a single NICU and the time of surveillance between the first and last patient.

Group 1 consisted of the old participants with voluntary participation. They started patient surveillance between January 2000 and December 2002 and participated continuously for 3 years (1095 days) in NEO-KISS. 26 NICUs met this criterion.

Group 2 consisted of the new participants with mandatory participation. They started with patient surveillance between January and December 2006 and participated continuously for 3 years. 95 NICUs met this criterion.

### Influence on infection rates

To answer the first question, data from 2007 were examined. NICU parameters and infection rates of the NICUs in both groups were compared by Chi-square or Wilcoxon rank sum test.

### Influence on surveillance effect

To stimulate further infection control measures, all NICUs participating received a biannual feedback report including crude and standardized infection rates of their own NICU compared with the national reference data until 2006. From 2007 on, a web-based data entry with the possibility of immediate data calculation and feedback has been employed. Problems of surveillance were discussed and prevention activities were shared at annual workshops. Because primary BSI is the most frequent HCAI in VLBW infants, the influence of participation in the surveillance system on HCAI rates focused on the incidence density of primary BSI. In an earlier investigation of 24 units during their first three years of continuous participation, a significant decrease in BSI between first and third year of participation was found [3].

For the second question, all VLBW-infants within the first three years of participation in both groups were considered. In the univariable analysis, primary BSI incidence densities in the individual years of participation were determined and compared. The relative risks (RR) and their 95% confidence intervals (CI95) were calculated. In the multivariable analysis, logistic regression and Poisson regression models were performed to identify significant risk factors for the occurrence of BSI. The following parameters were considered: birth weight (5 categories, 250 g steps), gestational age (4 categories, <27/27-28/29-30/>30 weeks), sex, mode of delivery (sectio, emergency sectio), multiple birth, surveillance end point (3 categories: 1800 g/transfer/died), and VLBW-volume year 2007 (≤/>30 VLBW infants). In the Poisson regression model, the log number of patient days was treated as offset parameter for number BSI. All parameters were considered in a full model and model parameters were excluded stepwise by the smallest chi-square value and p ≥ 0.05 in the type III test. The adjusted effect measures for the year of participation were calculated by generalized estimating equation (GEE) models that consider cluster effects within a NICU. In this model, all significant parameters from the first model building step were included. However, for face validity reasons, we added sex into all final models. The quasi-likelihood information criterion (QIC) as a modification of the Akaike information criterion (ACI) was used as goodness-of-fit measure in the GEE model. *P* values of less than 0.05 were considered significant. All analyses were performed with SPSS (IBM SPSS Statistics; IBM Corporation, Armonk, NY, USA) and SAS (SAS Institute Inc., Cary, NC, USA).

Our study was based on surveillance data. All data were anonymous and collected in accordance with the German http://recommendations of good epidemiological praxis with respect to data protection. As a federal law, the German Protection against Infection Act (Infektionsschutzgesetz §23) regulates the prevention and management of infectious disease in humans. All hospitals are obliged to collect and analyse continuously nosocomial infections and resistant pathogens. These routine data were reported to the National Reference Centre of the Surveillance of Nosocomial Infections. Ethical approval and informed consent were thus not required.

## Results

**Figure 1:**
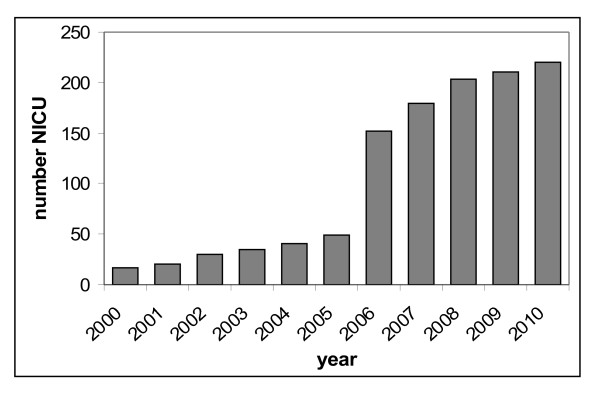
Development of the annual number of participating neonatal intensive care units (NICU) in NEO-KISS.

From the beginning in 2000 until 2005, a constant increase of the number of participating neonatal units was observed during the period of voluntary participation. Almost all remaining German neonatal ICUs caring for VLBW infants joined NEO-KISS after the introduction of mandatory participation. Figure 1 gives an overview about the annual number of participating neonatal units. An increase from 49 units participating in 2005 to 152 in 2006 was observed after the introduction of mandatory participation. At present, 220 centers submit data to NEO-KISS.

### Influence on infection rates

Table 1 shows NICU characteristics; distribution of very low birth weight (VLBW) infants according to birth weight groups; device utilization rates; and nosocomial infection and NEC incidence rates in the groups of old (voluntary participation) and new participants (mandatory participation) for the period from January to December 2007. A total of 4280 VLBW infants was considered for this analysis, with 1527 from the old participants and 2753 from the new participants. NICU characteristics differ significantly. Old participants have lower mean birth weight, have more VLBW infants per year, higher numbers of NICU beds and transfer patients more frequently before achieving 1800 g. However, healthcare-associated incidence densities rates are similar, with the exception of a significantly lower primary BSI rate in the birth weight group of 500-999 g in the group of new participants. Device-associated infection rates differ accordingly.

**Table 1:** Characteristics of NICU surveillance data in 26 old/voluntary and 95 new/mandatory participants in NEO-KISS, year 2007

**Parameter**	**Old/voluntary participants (N = 26, starting in NEO-KISS 1January 2000- December 2002)**	**New/mandatory participants (N = 95, starting in NEO-KISS January-December 2006)**	**p-value**^**a**^
NICU characteristics			
Number of beds in the NICU, median	24	16	**0.004**
Number of VLBW infants year 2007, median	53	23	**<0.001**
Months of participation until December 2010, median	123	56	**<0.001**
Number VLBW infants	1527	2753	
Birth weight (gram), mean	1100	1140	**0.001**
End of surveillance			
Transfer before 1800 g	21.4%	13.10%	
End of surveillance 1800 g	71.9%	80.0%	
Died	6.7%	7.0%	
Number VLBW infants according to birth weight groups (%)			
< 500 g	52 (3.4%)	92 (3.3%)	
1000-1499 g	615 (40.3%)	1012 (36.8%)	
1000-1499 g	860 (56.3%)	1649 (59.9%)	
Device utilization rates per 100 patient days (pooled mean)#			
CVC			
500-999 g	28.2	30.2	0.484
1000-1499 g	13.8	16.9	0.934
Tube			
500-999 g	21.2	21.2	0.267
1000-1499 g	6.3	6.1	0.676
Healthcare associated infection rates per 1000 patient days (pooled mean)#			
Primary BSI			
500-999 g	6.5	5.2	**0.023**
1000-1499 g	3.1	3.2	0.964
Pneumonia			
500-999 g	1.0	0.8	0.286
1000-1499 g	0.4	0.1	0.053
Device-associated infection rates per 1000 device days (pooled mean)#			
CVC-BSI			
500-999 g	11.4	9.4	**0.011**
1000-1499 g	7.7	6.9	0.150
Tube associated pneumonia			
500-999 g	2.2	2.3	0.634
1000-1499 g	3.9	0.4	**0.010**

NICU, neonatal intensive care unit; BSI, bloodstream infection; CVC-BSI, Central venous catheter-associated bloodstream infection; VLBW, very low birth weight; # Data for the birth weight group < 500 g are not shown because of the small number of VLBW infants in this group; ^a^ p-value, Wilcoxon test; ^b^ p-value, Chi square test.

### Influence on surveillance effect

Table 2 describes the development of NICU characteristics and BSI rates during the first three years of participation in the surveillance system in both groups. The univariable comparison of primary BSI incidence density reveals a relative risk of 0.79 (CI95 0.68-0.91) in the first group and of 0.79 (CI95 0.69-0.90) in the second group also. Using multivariate analysis with the endpoint incidence density of primary BSI leads to an adjusted incidence rate ratio (IRR) of 0.78 (CI95 0.66-0.93) for the first group and 0.81 (CI95 0.68-0.97) for the second group (Table 3).

**Table 2:** Development of primary BSI rates during the first three years of participation in 26 old/voluntary and 95 new/mandatory participants in NEO-KISS

	**Old/voluntary participants (N = 26, starting in NEO-KISS January 2000- December 2002)**				**New/mandatory participants (N = 95, starting in NEO-KISS January-December 2006)**			
	1^st^ year	2^nd^ year	3^rd^ year	Total	1^st^ year	2^nd^ year	3^rd^ year	Total
Number of VLBW infants	1421	1310	1207	3938	2855	2740	2676	8271
Pooled mean birth weight (g)	1070	1125	1085	1095	1145	1140	1146	1140
Number of patient days	59314	52112	48651	160077	103198	100382	98416	301996
Mean observation days per patient	41.7	39.8	40.3	40.6	36.1	36.6	36.8	36.5
CVC utilization rates (%)	21.2	22.1	22.6	21.9	24.9	24.2	22.7	24.0
Number of BSI	456	347	294	1097	509	439	384	1332
BSI Incidence density (per 1000 patient days)	7.7	6.7	6.0	6.9	4.9	4.4	3.9	4.4

NICU, neonatal intensive care unit; BSI, bloodstream infection; VLBW, very low birth weight.

**Table 3:** **Results of univariable and multivariable analysis comparing primary BSI rates the 3**^**rd **^**and 1**^**st **^**year of participation in NEO-KISS**

**HCAI infection rate**	**Old/voluntary participants (N = 26, starting in NEO-KISS January 2000- December 2002)**		**New/mandatory participants (N = 95, starting in NEO-KISS January-December 2006)**	
	univariable analysis pooled data 3^rd^*vs.* 1^st^ year	multivariable analysis adjusted effect measures	univariable analysis pooled data 3^rd^*vs.* 1^st^ year	multivariable analysis adjusted effect measures
BSI Incidence density (per 1000 patient days)	RR = 0.79; (CI95 0.68-0.91); p = 0.001	IRR^a^ = 0.78; (CI95 0.66-0.93); p = 0.005	RR =0.79; (CI95 0.69-0.90); p < 0.001	IRR^a^ = 0.81; (CI95 0.68-0.97); p = 0.019

CI95, 95% confidence interval; RR, relative risk; IRR, adjusted incidence rate ratio; p, p-value;

^a^ Poisson regression models with the outcome number BSI and log number patient days as offset parameter calculated by generalized estimating equation (GEE) model which account for clustering effect within a single NICU, consider the following parameters: birth weight (5 categories, 250gram steps), gestational age (4 categories, <27/27-28/29-30/>30 weeks), sex, mode of delivery (sectio) and surveillance endpoint (3 categories: 1800 g/transfer/died).

## Discussion

The transition from a voluntary to a mandatory surveillance system did not lead to lower quality of reference data and also did not influence the use of surveillance data for reducing nosocomial infections, at least in the field of neonatal intensive care medicine [4]. Even those NICUs not interested in participating in a voluntary surveillance system were able to record HCAI with a similar sensitivity and specificity and were also able to use the data for reducing HCAI rates to the same extent as NICUs with voluntary participation and therefore probably more interest in this subject. This is interesting because one could argue that NICUs participating on a voluntary basis are more likely to be interested and enthusiastic about quality improvement than those who are forced to gather and report HCAI data. Accordingly, volunteers might be more amenable to participation in training and validation exercises, produce higher quality data and achieve better patient outcomes [5]. But this seems not to be the case. After starting surveillance on a mandatory basis, the healthcare workers responsible for surveillance seem to be as interested and as stimulated as those in units with an interest from the beginning.

However, the data of this mandatory surveillance system are still confidential. Only the head of each department receives their data together with the reference data and can decide how to use the information. Many countries have meanwhile introduced mandatory participation in combination with public reporting instead of voluntary participation and confidential data feedback. For example, many US states use the NHSN system as a surveillance tool for public reporting. A comparison of NHSN data from 2006 for neonatal ICUs with the data for 2010 shows a significant decrease of primary BSI rates from many HCAI rates can be found (from 5.2 to 2.3 in the birth weight group 751-1000 g and from 3.4 to 1.4 in the birth weight group 1001–1500, Level II/III) [6], [7]. However, it remains an open question if this is due to real success in decreasing HCAI rates or due to underreporting because of public reporting. Therefore it seems to be interesting that the process from a voluntary to a mandatory system alone was not associated with decreased reference data quality.

### This analysis has some limitations

Both groups are not really comparable, because many structural parameters differed between both groups. It may be that larger centers that transfer more patients after the critical period had a higher interest from the beginning in participating in a surveillance system. These differences may also explain the significantly lower BSI rates in the birth weight group 500–999 g.

The comparison of HCAI rates in old and new participating NICUs is biased by the fact that at least 26 of the old participants have already achieved a significant decrease of BSI rates in their first three years of participation.

The reduction of BSI rates during the observation periods may result not only from the surveillance effect but also from medical and technological achievements in this time period.

In conclusion, the step from a voluntary to a mandatory HCAI surveillance system alone may lead to substantial improvements. Awareness of HCAI increases in all comparable institutions, and HCAI identification skills are improving on a countrywide scale. Employing infection rate calculation and interpretation to stimulate infection control activities has also improved.

## Competing interests

The authors declare that they have no competing interests.

## Author’s contribution

PG, CG and BP are responsible for the concept, design and implementation of the NEO-KISS module. FS performed the statistical analysis and interpreted the data. PG drafted the manuscript. CG, BP and FS critically revised the manuscript. All authors read and approved the final manuscript.
